# The Treatment of Chorea with Rheumatism Phylacogen

**Published:** 1913-05

**Authors:** E. Bates Block

**Affiliations:** Professor of Nervous Diseases, Atlanta College of Physicians and Surgeons, Atlanta, Ga.


### Journal-Record of Medicine

Successor to Atlanta Medical and Surgical Journal, Established 1855
and Southern Medical Record, Established 1870

**OWNED BY THE ATLANTA MEDICAL JOURNAL COMPANY**

PUBLISHED MONTHLY

*Official Organ Fulton County Medical Society, State Examining Board, Presbyterian Hospital, Atlanta, Birmingham and Atlantic Railroad Surgeons' Association, Chattahoochee Valley Medical and Surgical Association, Etc.*

EDGAR BALLENGER, M. D., Editor
BERNARD WOLFF, M. D., Supervising Editor
A. W. STIRLING, M. D., C. M., D. P. H.; J. S. HURT, B. Ph., M.D.
GEO. M. NILES, M. D., W. J. LOVE, M. D., (Ala.); Associate Editors
E. W. ALLEN, Business Manager

**COLLABORATORS**

DR. W. F. WESTMORLAND, General Surgery
F. W. McRAE, M. D., Abdominal Surgery
H. F. HARRIS, M. D., Pathology and Bacteriology
E. B. BLOCK, M. D., Diseases of the Nervous System
MICHAEL HOKE, M. D., Orthopedic Surgery
CYRUS W. STRICKLER, M. D., Legal Medicine and Medical Legislation
E. C. DAVIS, A. B., M. D., Obstetrics
E. G. JONES, A. B., M. D., Gynecology
R. T. DORSEY, JR., B. S., M. D., Medicine
L. M. GAINES, A. B., M. D., Internal Medicine
GEO. C. MIZELL, M. D., Diseases of the Stomach and Intestines
L. B. CLARKE, M. D., Pediatrics
EDGAR PAULIN, M. D., Opsonic Medicine
THEODORE TOEPEL, M. D., Mechano Therapy
R. R. DALY, M. D., Medical Society
A. W. STIRLING, M. D., Etc., Diseases of the Eye, Ear, Nose and Throat
BERNARD WOLFF, M. D., Diseases of the Skin
E. G. BALLENGER, M. D., Diseases of the Genito-Urinary Organs

VOLUME LX — MAY, 1913 — No. 2

### THE TREATMENT OF CHOREA WITH RHEUMATISM PHYLACOGEN

BY E. BATES BLOCK M. D.

Professor of Nervous Diseases, Atlanta College of Physicians and Surgeons, Atlanta, Ga.

It is well known that some Chorea is due to the same micro-organism that produces rheumatism, and the frequent association of some of the rheumatic manifestations with

Chorea is a common observation. At least sixty per cent, or more, of the cases of Chorea have rheumatism within a period of three years, before or three years after the attack of Chorea. Nevertheless some uncomplicated cases of Chorea occur, just as some uncomplicated cases of rheumatism occur, but as the same time it is quite common to find a slight cardiac murmur, or other evidences of rheumatism with Chorea.

As we know, Chorea shows a tendency to get well in a few weeks or a few months, usually whether anything is done for it or not, so that any method of treatment renders itself liable to the criticism that it may have gotten well even if this treatment had not been carried out. Still more is this paper liable to criticism, for the fact that the cases received other treatment at the same time that they received the Rheumatism Phylacogen. Nevertheless, the treatment with Phylacogen was followed so rapidly and steadily by improvement and ultimately by a cessation of symptoms, that I feel convinced in my own mind that the rheumatism phylacogen bears the same relation to the treatment of Chorea that it does to the treatment of rheumatism.

In the first case which I reported the treatment with Arsenic and the usual anti-rheumatic drugs was of little benefit, while the use of the phylacogen was quickly followed by improvement and eventually recovery. In regard to dosage, I believe the excessive doses usually recommended are entirely too severe in effect, and unnecessary in results, to be given, and that the same good can be accomplished by the smaller doses, given for a longer period of time with far less distress to the patient, although I think the total quantity should amount to 60 Cubic Centimeters.

CASE 1

J. L., a boy 7 years old was seen first on May 5th, 1912.

Family History—Father and Mother living and well. The patient was an only child, except a half brother who was living and well. His mother had had one miscarriage. There had been much rheumatism in his mother's family and his mother has it slightly at times. No nervous troubles in the family.

Past History—Never had any diseases before. Tonsils were removed three years ago.

Present Illness—Began with rheumatism in ankle and knee three months before this trouble began, December 1912. He was very fidgity, had twitching and jerking all over, but never had any mental symptoms. Later he had rheumatism in his ankles, wrists and fingers, and his ankles were swollen about Christmas. The examination of his heart showed a Mitral systolic murmur, transmitted throughout the axilla, and a softer murmur over the body of the heart and at the base. The urine showed a specific gravity of 1010 to 1022, and a sediment of amorphous urates, but was otherwise normal. His leucocyte count was 11,000 and the red blood cells 4,310, 000. His temperature was 100 on admission but was about normal after that. He was treated then with Sodium Salicylate, and sodium bicarbonate, Fowler's Solution, Ergot, Sodium Bromide, laxatives, Aspirin, hot and cold fomentations to the spine. Tr. Nux Vomica, warm tub baths, diet excluding meats, eggs and tomatoes, but in spite of these he continued to have excessive arhythmic, inco-ordinate, involuntary movements, and had to be mechanically restrained to prevent his falling out of the bed. Treatment was begun on May 5th, 1912, with very little effect and on June 22nd, treatment was started with Rheumatism Phylacogen 10 Minims subcutaneously. This was given then daily, and each dose increased 5 minims. On June 25th he was not fidgity at all and there was practically no fidgitiness after this. When the dose reached 50 minims, he had a raise of temperature to 104 3-5 and was slightly delirious, so the dose was reduced to forty minims and was given every second day. This did not produce a rise in temperature above 99-1-5 to 100. The patient complained of Rheumatic pains throughout his illness till after the third injection of phylacogen, after which there were no pains. The phylacogen was increased to 45 minims on July 6th and did not produce any febrile reaction, so was continued at that dose. On July 11th, his hands jerked slightly. The highest dose of phylacogen given was 50 minims and the total amount 60 Cubic Centimeters, and the last dose was given on July 20th. The patient was entirely well after this of his Chorea and rheumatism and heart was greatly improved and he left for home on July 25th. He was seen again on Feb. 10, 1913. He had had no return of Chorea or rheumatism, but still had a mitral regurgitation as a result of his rheumatic endocarditis, although it had improved far beyond my expectations and he was able to go to school and play with moderate exertion without shortness of breath or oedema of the ankles. He was not allowed to run.

CASE 2

R. T., a girl 8 1-2 years, was first seen on July 2nd, 1912.

Family History—Her mother's mother's Father had had rheumatism.

Past History—She had had pains in her legs, ankles and knees on and off for four or five weeks but never had any other diseases.

Present Illness—Began with laughing and crying easily and very sensitive disposition for four or five months. She became fidgity a week ago, it began in the right leg, and then the left leg became affected, then the left arm and then the right arm. It was worse in the left arm than anywhere else. The face, neck and head were also affected. She had been losing weight for a month. There were no mental symptoms except a marked emotional instability. Her tongue was very fidgity and there was difficulty in swallowing. She had much trouble in talking and could not usually be understood at all, and rarely attempted to say more than one word at a time. There was no sore throat. There was a slight systolic murmur at the apex and over the body of the heart lying down, but this was not transmitted into the axilla and was not heard sitting up. The muscular movements were extreme and she had to be assisted in walking. There was no strength in the left arm and it was practically useless. She was given Fowler's Solution and on July 3rd, 1913, Rheumatism Phylacogen was started. She was given 5 minims the first day and increased 5 minims every day, skipping Sundays, till she took 60 minims at a dose. On July 22nd, the improvement was very noticeable both in talking and fidgetiness. On Aug. 2nd the speech was normal and the Chorea scarcely noticeable, and by Aug. 9th, was entirely gone. The phylacogen was stopped on Aug. 16th, after she had taken 60 Cubic Centimeters. The strength in her arm returned and the rheumatism and Chorea entirely disappeared.

CASE 3

G. M., a girl aged 10 1-2 years was seen on May 29th, 1912.

Present History—She began two weeks ago to twitch and fidget. Her tonsils were slightly enlarged and her action was irregular in force and rhythm, with a slight systolic murmur at the apex, which was not transmitted outward and was not constant. She was given Fowler's Solution with benefits for a while, when her fidgetiness became worse again about July 4th, so rheumatism phylacogen was started on July 9th, starting with 5 minims and increasing 5 minims daily, omitting Sunday, until 50 minims were given at each dose. Her Chorea gradually subsided and disappeared entirely. She was given in all 30 minims of Rheumatism Phylacogen; the last dose was given July 29th, 1912. There was no evidence of Rheumatism in this case and no previous history of it. This was a mild case of Chorea with some emotional instability.

CASE 4

L. M., a girl aged 10 years was seen first on Nov. 9th, 1911.

She has always been nervous. Had chicken-pox in December 1911.

Present Illness—Her mother noticed two weeks ago that her mind seemed a little blank and her fingers and feet twitched irregularly and jerk and she dropped things often. She was very fidgety. Not emotional. She had no rheumatism or growing pains. Her tonsils were removed at 4 years of age. She was given Fowler's Solution and her fidgetiness was gone by Dec. 5th, 1911. She then had an attack of chicken-pox in Dec. and on Dec. 28th, had rheumatism, which lasted three or four days, in her right knee and left ankle, and she became cross and irritable. This disappeared under diet, colon lavage, and Aspirin. There was some return of fidgetiness which stopped however, by Jan. 23rd, 1912., but returned off and on in Feb. and March and on March 23rd, had an attack of cystitis. She did well after this, but she returned to me on Nov. 5th, and stated that two weeks ago the fingers on both hands began to move irregularly and had rheumatism in the knees and ankles in September and October. Rheumatism phylacogen was started on Nov. 6th, giving 5 minims daily except Sundays, and increasing 5 minims each day up to 25 minims. On Dec. 14th she had rheumatism in left knee and shoulders, and on Jan. 2nd, in both hips and knees. The phylacogen was stopped on Jan. 16th and there had not been any nervousness, pains or aches for a week and none since then. She was given 60 Cubic Centimeters of phylacogen. She took Aspirin and Fowler's Solution as well as phylacogen, while under treatment.

